# First-phase ejection fraction by cardiovascular magnetic resonance predicts outcomes in aortic stenosis

**DOI:** 10.1186/s12968-021-00756-x

**Published:** 2021-06-10

**Authors:** Haotian Gu, Rong Bing, Calvin Chin, Lingyun Fang, Audrey C. White, Russell Everett, Nick Spath, Eunsoo Park, John B. Chambers, David E. Newby, Amedeo Chiribiri, Marc R. Dweck, Phil Chowienczyk

**Affiliations:** 1 British Heart Foundation Centre of Research Excellence, King’s College London, London, UK; 2 British Heart Foundation Centre for Cardiovascular Science, University of Edinburgh, Edinburgh, UK; 3 Department of Cardiology, National Heart Centre, Singapore, Singapore; 4 Department of Ultrasound, Union Hospital, Tongji Medical College, Huazhong University of Science and Technology, Wuhan, China; 5 Edinburgh Heart Centre, Royal Infirmary of Edinburgh, Edinburgh, UK; 6 Cardiothoracic Centre, Guy’s and St Thomas’s Hospitals, London, UK; 7 Department of Clinical Pharmacology, St Thomas’ Hospital, London, SE1 7EH UK

**Keywords:** First-phase ejection fraction, Cardiovascular magnetic resonance, Echocardiography, Aortic stenosis

## Abstract

**Background:**

First-phase ejection fraction (EF1; the ejection fraction measured during active systole up to the time of maximal aortic flow) measured by transthoracic echocardiography (TTE) is a powerful predictor of outcomes in patients with aortic stenosis. We aimed to assess whether cardiovascular magnetic resonance (CMR) might provide more precise measurements of EF1 than TTE and to examine the correlation of CMR EF1 with measures of fibrosis.

**Methods:**

In 141 patients with at least mild aortic stenosis, we measured CMR EF1 from a short-axis 3D stack and compared its variability with TTE EF1, and its associations with myocardial fibrosis and clinical outcome (aortic valve replacement (AVR) or death).

**Results:**

Intra- and inter-observer variation of CMR EF1 (standard deviations of differences within and between observers of 2.3% and 2.5% units respectively) was approximately 50% that of TTE EF1. CMR EF1 was strongly predictive of AVR or death. On multivariable Cox proportional hazards analysis, the hazard ratio for CMR EF1 was 0.93 (95% confidence interval 0.89–0.97, *p* = 0.001) per % change in EF1 and, apart from aortic valve gradient, CMR EF1 was the only imaging or biochemical measure independently predictive of outcome. Indexed extracellular volume was associated with AVR or death, but not after adjusting for EF1.

**Conclusions:**

EF1 is a simple robust marker of early left ventricular impairment that can be precisely measured by CMR and predicts outcome in aortic stenosis. Its measurement by CMR is more reproducible than that by TTE and may facilitate left ventricular structure–function analysis.

## Background

In conditions where there is impairment of myocardial contractile function early in systole, a length-dependent activation of the myocyte may preserve ejection fraction (EF) at the expense of slower but sustained contraction. This may explain why traditional measures of “systolic function” such as EF and newer measures such as global longitudinal strain (GLS) that measure total contraction over systole may be insensitive in detecting early systolic dysfunction. We have previously demonstrated that first-phase ejection fraction (EF1) as measured by transthoracic echocardiography (TTE), the EF measured up to the time of maximal aortic flow velocity on continuous wave Doppler which corresponds to active systole, is a more sensitive measure than EF or GLS in detecting early systolic dysfunction. EF1 as measured by TTE is of prognostic value in patients with aortic stenosis (AS) [1, 2], the most common form of primary heart valve disease [3]. Although TTE is the most commonly used diagnostic tool for evaluation of LV function in patients with AS, cardiovascular magnetic resonance (CMR) imaging is the gold standard imaging modality for assessing the myocardium, in particular for the quantification of left ventricular (LV) volumes and function, and tissue characterization [4, 5]. Furthermore, the use of CMR facilitates a greater degree of functional-structural correlation, potentially providing insight into mechanisms impairing early systolic function. The objective of the present study was to assess whether CMR might provide more precise measurements of EF1 than TTE and to examine the correlation of CMR EF1 with myocardial fibrosis, afterload and clinical outcomes.

## Methods

### Patient population

This was a retrospective analysis of an observational cohort of patients with at least mild AS (peak aortic velocity ≥ 2 m/s) who were prospectively recruited from the Edinburgh Heart Centre between March 2012 and August 2014 [6]. We recently reported clinical and prognostic associations of TTE EF1 in this cohort [2]. Exclusion criteria were other valvular heart disease (greater than mild severity), comorbidities with limited life expectancy, contraindications to gadolinium, and acquired or inherited non-ischaemic cardiomyopathies as assessed by history or CMR. Patients underwent clinical evaluation, venous blood sampling for plasma concentrations of high-sensitivity cardiac troponin I (hs-cTnI, ARCHITECT STAT assay, Abbott Laboratories, Abbott Park, Illinois, USA) and brain natriuretic peptide (BNP, Triage assay, Biosite Inc., San Diego, California, USA), electrocardiography, TTE and CMR. Referral for aortic valve intervention was undertaken by the treating cardiologist in accordance with routine practice and contemporary guidelines [2]. The study was approved by the regional ethics committee (10/S1102/24) and conducted in accordance with the Declaration of Helsinki. Written informed consent was obtained from all subjects. The primary outcome was a combination of aortic valve replacement (AVR) and death (identified through medical records and the General Register of Scotland).

### Cardiovascular magnetic resonance and EF1

Detailed CMR protocols in this cohort have been described [6]. All baseline scans were performed on a 3 T CMR scanner (MAGNETOM Verio, Siemens Healthineers, Erlangen, Germany). Late-gadolinium enhancement (LGE) imaging was performed 15 min after intravenous administration of 0.1 mmol/kg gadobutrol and independently assessed by two investigators (CC and MD). T1 mapping was performed using the modified Look-Locker inversion recovery sequence [2]. Native T1, extracellular volume fraction (ECV%) and indexed extracellular volume (iECV = ECV% x LV diastolic myocardial volume indexed to body surface area) were measured [2, 6, 7]. Both ECV% and iECV included non-infarct and excluded infarct LGE [2]. GLS was measured using cvi42 (Circle Cardiovascular Imaging, Calgary, Alberta, Canada). A fully automated strain analysis was carried out with two-dimensional GLS as the primary assessment.

**Fig. 1:**
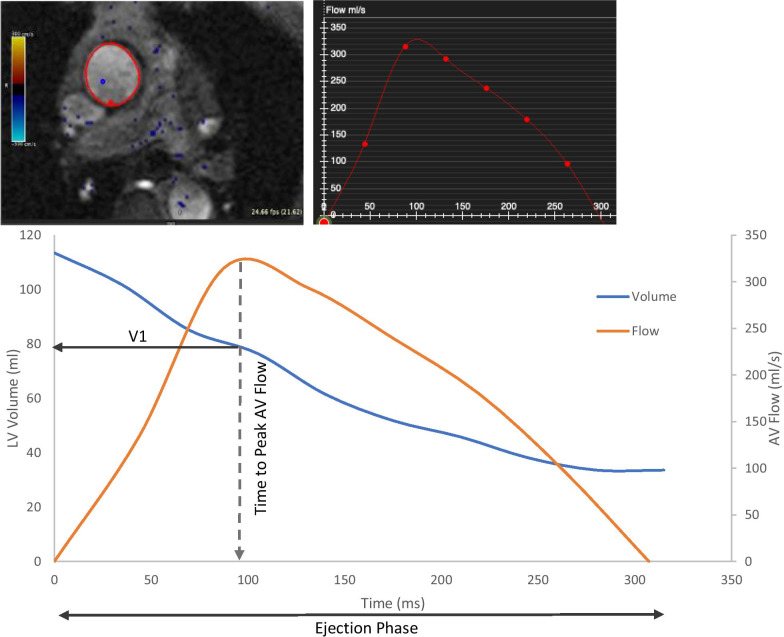
Representative cardiovascular magnetic resonance (CMR) images and measures of aortic valve flow and left ventricular (LV) volume from a patient in the series. Left ventricular volume was measured from CMR short-axis stack 3D, aortic valve flow was measured from phase contrast flow images and time to peak aortic valve flow was derived from the aortic valve flow-time curve. First-phase ejection fraction (EF1) was then calculated using equation: EF1 = (EDV-V1)/EDV%, where EDV is end-diastolic volume and V1 is volume at time of peak aortic valve flow

CMR EF1 was retrospectively analysed by one observer (HG) blinded to patient characteristics and outcomes using cvi42. Time to peak aortic valve flow was derived from the phase contrast aortic valve flow-time curve (Fig. 1). LV volumes were measured using short-axis 3D stack following the recommendations of the Society for Cardiovascular Magnetic Resonance [8].

CMR EF1 was then calculated using the following equation [1]:

$${\text{EF1}}\, = \,\left( {{\text{EDV}} - {\text{V1}}} \right) \,/{\text{EDV }}\%$$

where EDV is end-diastolic volume and V1 is LV volume at the time of peak aortic flow (Fig. 1). V1 was measured from the frame closest to peak aortic velocity estimated from the number of frames per unit time multiplied by the time to peak aortic velocity. We also examined a similar metric to EF1 that can be derived entirely from the integral of aortic flow over time: aortic flow-derived volume up to the time of peak flow (SV1) expressed as a ratio of aortic flow-derived volume over systole (SV). In the absence of mitral regurgitation SV1/SV is theoretically equal to EF1/EF and avoids the limitation of phase contrast and cine imaging not being measured concurrently.

Intra- and inter-observer variability in measurements of CMR EF1 was assessed in 40 randomly selected subjects by two observers by Bland–Altman analysis calculating the SD of the difference between readings and coefficient of variation defined as the SD expressed as a percentage of the mean measurement.

### Transthoracic echocardiography and EF1

TTE was performed in all patients according to American Society of Echocardiography guidelines [9]. The detailed protocol and measurement of EF1 by TTE have been previously described [2]. AS severity was defined by aortic valve area (AVA) and mean pressure gradient (MPG) according to current guidelines [10, 11]. Valvuloarterial impedance (Zva = [systolic blood pressure + mean aortic valve gradient]/stroke volume), a validated prognostic measure of global LV afterload in AS, was also calculated and indexed to body surface area [12].

### Statistical analysis

Continuous variables were tested for normality with the Shapiro–Wilk test and are presented as median [interquartile range (IQR)] or mean ± standard deviation (SD). Non-normally distributed continuous variables were log_2_-transformed for regression models. CMR GLS was log_2_-transformed after addition of a constant (greatest GLS + 1). The Pearson correlation coefficient and Bland–Altman analysis were used for comparing CMR and TTE EF1. Intra- and inter-observer variability for CMR EF1 and TTE EF1 was assessed by the standard deviations of differences and by Bland–Altman plots.

CMR EF1 was dichotomized using a previously defined cut-off value derived from our previous work using TTE, adjusted for the mean difference between values of CMR and TTE EF1. Univariable and multivariable linear regression modelling was performed to identify the associations between CMR EF1 and relevant clinical and CMR variables including age (per decade), male sex, Zva, infarct LGE, iECV, hs-cTnI and brain natriuretic peptide (BNP). These models were constructed with haemodynamic and CMR parameters followed by the addition of biochemistry and clinical parameters. Infarct LGE and iECV are conceptually distinct assessments and as such were chosen over other fibrosis measures. Cumulative event rates were examined using Kaplan–Meier curves for the combined primary outcomes of AVR or all-cause mortality. Assumptions for proportionality were checked and the time-dependent association between CMR EF1 and the primary outcome assessed using Cox proportional hazard models, with the covariables of age (per decade), male sex, New York Heart Association (NYHA) dyspnoea class, MPG, EF1, infarct LGE and iECV. Two-sided *p*-values < 0.05 were considered statistically significant. Analysis was performed using R version 3.5.0 (R Foundation for Statistical Computing, Vienna, Austria).

## Results

### Patient characteristics and major events

**Fig. 2:**
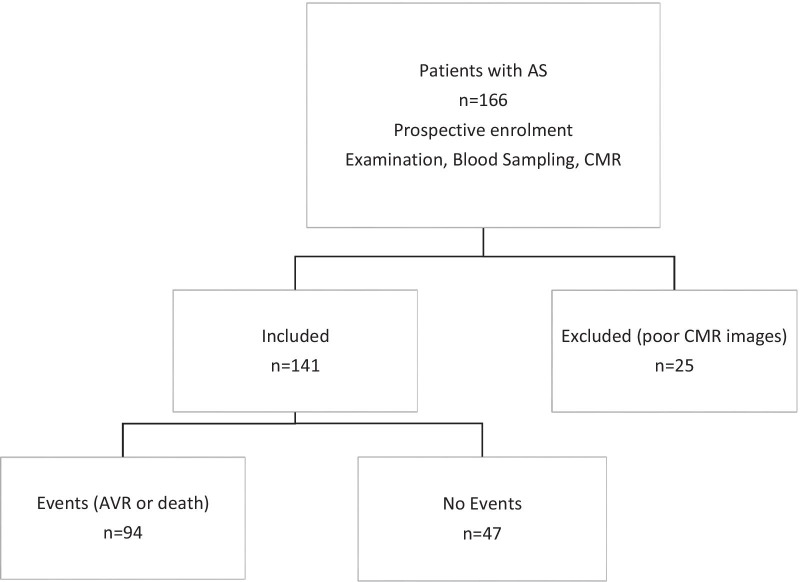
Study flow chart

Of the 166 patients in the original cohort, 25 were excluded due to suboptimal CMR images (Fig. 2). Thus, 141 patients, including 28 with mild, 41 with moderate, and 72 with severe AS were included in the final analysis. Twenty-five patients classified as severe AS due to an aortic valve area < 1 cm^2^ had a mean gradient < 40 mmHg. Ninety-four patients experienced an event with a median time to event of 13.3 (IQR: 2.0 to 36.1) months, including 78 who had AVR and 16 who died without AVR. Patients with reduced (≤ 20%) CMR EF1 (n = 74) were of similar age but had worse NYHA class, more severe AS than those with preserved CMR EF1 (> 20%) (Table 1). There was no difference in EF or GLS between those with reduced or preserved CMR EF1 (Table 1).

**Table 1:** Basic characteristics

Characteristic	Overall N = 141	CMR EF1 ≤ 20%N = 74^1^	CMR EF1 > 20%N = 67^1^	*p*-value^2^
Age	70 (63, 75)	70 (64, 75)	69 (62, 75)	0.5
Sex				0.5
Female	44 (31%)	25 (34%)	19 (28%)	
Male	97 (69%)	49 (66%)	48 (72%)	
Hypertension	94 (67%)	51 (69%)	43 (64%)	0.6
Dyslipidaemia	66 (47%)	36 (49%)	30 (45%)	0.6
Diabetes	22 (16%)	9 (12%)	13 (19%)	0.2
Coronary artery disease	53 (38%)	30 (41%)	23 (34%)	0.4
Systolic BP (mmHg)	147 (133, 162)	145 (132, 162)	148 (137, 163)	0.5
Diastolic BP (mmHg)	83 (76, 92)	84 (77, 92)	82 (76, 92)	0.5
NYHA class				** < 0.001**
I	66 (47%)	22 (30%)	44 (66%)	
II	45 (32%)	26 (35%)	19 (28%)	
III	27 (19%)	23 (31%)	4 (6%)	
IV	3 (2%)	3 (4%)	0 (0%)	
AV Vmax (m/s)	3.8 (3.3, 4.4)	4.2 (3.8, 4.7)	3.4 (2.7, 3.8)	** < 0.001**
AV MPG (mmHg)	33 (22, 43)	41 (32, 48)	24 (15, 33)	** < 0.001**
AVA (cm^2^)	0.87 (0.73, 1.08)	0.78 (0.66, 0.91)	1.04 (0.81, 1.31)	** < 0.001**
Zva (mmHg/mL/m^2^)	4.01 (3.26, 4.47)	4.11 (3.39, 4.78)	3.91 (3.25, 4.23)	**0.035**
LVMI (g/m^2^)	87 (73, 101)	90 (74, 103)	86 (72, 96)	0.15
SVI (ml/m^2^)	47 (41, 55)	48 (41, 56)	47 (40, 53)	0.6
CMR EF (%)	67 (64, 71)	67 (63, 71)	67 (64, 71)	0.5
CMR EF1 (%)	19 (14, 23)	14 (9, 17)	24 (22, 26)	** < 0.001**
TTE EF1 (%) (n = 126)	25 (18, 30)	20 (12, 28)	27 (25, 30)	** < 0.001**
CMR GLS (%)	− 18.0 (− 20.1, − 15.9)	− 17.7 (− 19.3, − 15.3)	− 18.6 (− 20.5, − 16.1)	0.082
Native T1 (ms)	1179 (1155, 1206)	1190 (1162, 1216)	1171 (1153, 1195)	**0.046**
ECV fraction (%)	27.6 (25.7, 29.7)	27.7 (25.7, 29.8)	27.4 (25.8, 29.1)	0.4
iECV (ml/m^2^)	22 (18, 27)	23 (19, 29)	22 (18, 26)	0.10
Infarct LGE	21 (15%)	11 (15%)	10 (15%)	> 0.9
Non-infarct LGE	40 (28%)	23 (31%)	17 (25%)	0.5
hs-cTnI (ng/L)	7 (4, 12)	8 (4, 15)	5 (3, 10)	0.055
BNP (ng/L)	26 (11, 54)	29 (14, 71)	19 (9, 50)	0.13

^*1*^Statistics presented: median (IQR); n (%)

^*2*^Statistical tests performed: Wilcoxon rank-sum test; chi-squared test of independence; Fisher's exact test; values for* P* < 0.05 are shown in bold

*EF1* first phase ejection fraction, *SBP* systolic blood pressure, *DBP* diastolic blood pressure, *NYHA* New York Heart Association; *AV* aortic valve, *AVA* aortic valve area, *Zva* aortic impedance; *LVMI* left ventricular mass index; SVI stroke volume index; CMR cardiac magnetic resonance, *EF* ejection fraction, *GLS* global longitudinal strain, *ECV* extracellular volume, *iECV* indexed extracellular volume; *LGE* late gadolinium enhancement, *hs-cTnI* high sensitive cardiac troponin, *BNP* brain natriuretic peptide

### Comparison of CMR EF1 and TTE EF1

**Fig. 3:**
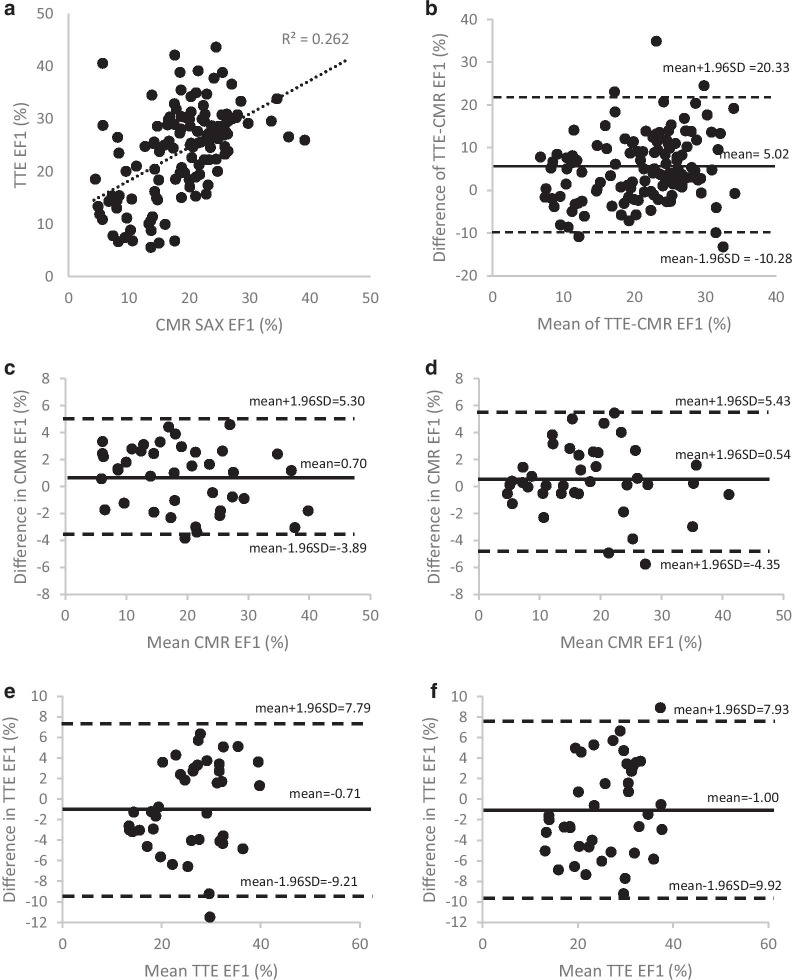
Pearson correlation (**a**) and Bland–Altman plot (**b**) for EF1 measured by transthoracic echocardiography (TTE) and CMR. Bland–Altman plot of CMR EF1 (**c**) intra-observer and (**d**) inter-observer variability; TTE EF1 (**e**) intra-observer and (**f**) inter-observer variability in 40 randomly selected subjects

Of the 141 patients in whom EF1 was measured by CMR, 126 had measurements of EF1 by both CMR and TTE with a median of 4 (IQR: 5) days between CMR and TTE. There was no significant difference in heart rate (65 ± 13 vs 64 ± 11 bpm, *p* = 0.286) on the CMR and TTE visit days. CMR EF1 correlated with TTE EF1 (r = 0.51, *p* < 0.001) but there was a small but significant systematic bias with mean ± SD difference between CMR EF1 and TTE EF1 of -5.0 ± 7.8% (Fig. 3a, b). Intra-and inter-observer variation for CMR EF1 was 2.3% and 2.5% units respectively for the SD of the difference between intra- and inter-observer readings giving coefficients of variation of 12.4% and 13.8% respectively (Fig. 3c, d). For TTE EF1, intra- and inter-observer variation was greater with SDs of 4.3% and 4.6% (each *p* < 0.01 compared to values by CMR) and coefficients of variation of 16.8% and 17.7% for intra- and inter-observer readings respectively (Fig. 3e, f). There was a relatively poor correlation between SV1/SV and CMR EF1/EF (R = 0.23, *p* = 0.006) and between SV1/SV and TTE EF1/EF (R = 0.17, *p* = 0.046).

### CMR EF1 and other clinical characteristics

CMR EF1 fell progressively with increasing severity of AS: mild 24.7 ± 4.5%, moderate 21.9 ± 7.3% and severe 14.5 ± 6.1% AS. On univariate linear regression, CMR EF1 correlated with mean gradient and Zva (Table 2). None of the measures of fibrosis were correlated with CMR EF1 and stepwise multivariate linear regression models using the pre-specified covariables (Table 3) demonstrated Zva (p < 0.05) to be independently associated with CMR EF1.

**Table 2:** Univariable linear regression models for CMR EF1

	Coefficient	95% CI	r^2^
Age per 10 years	− 0.08	− 0.18, 0.02	0.02
Male sex	0.01	− 0.24, 0.26	0.00
Hypertension	− 0.15	− 0.39, 0.10	0.01
Ejection fraction (log_2_)	0.43	− 0.33, 1.19	0.01
Mean gradient (log_2_)	− 0.47***	− 0.60, − 0.33	0.26
Left ventricular mass index (log_2_)	− 0.25	− 0.59, 0.08	0.02
Valvuloarterial compliance (log_2_)	− 0.36*	− 0.68, − 0.03	0.03
Native T1 (log_2_)	− 1.23	− 3.52, 1.05	0.01
Extracellular volume fraction (log_2_)	0.21	− 0.66, 1.08	0.00
Indexed extracellular volume (log_2_)	− 0.17	− 0.46, 0.12	0.01
Late gadolinium enhancement (any)	− 0.05	− 0.29, 0.19	0.00
Infarct late gadolinium enhancement (any)	− 0.12	− 0.44, 0.21	0.00
Global longitudinal strain (log_2_)	− 0.12	− 0.37, 0.12	0.01
High− sensitivity cardiac troponin I (log_2_)	− 0.05	− 0.13, 0.02	0.01
Brain natriuretic peptide (log_2_)	− 0.05	− 0.12, 0.01	0.02

***p < 0.001; *p < 0.05

**Table 3:** Stepwise multivariable linear regression models for CMR EF1

	Model 1 (r^2^ 0.06) n = 137			Model 2 (r^2^ 0.07) n = 117			Model 3 (r^2^ 0.07) n = 117		
	Coefficient	95% CI	*p* value	Coefficient	95% CI	*p* value	Coefficient	95% CI	*p* value
Zva	− 0.44	− 0.79, − 0.09	** < 0.05**	− 0.35	− 0.73, 0.04	ns	− 0.32	− 0.71, 0.08	ns
iECV	− 0.26	− 0.57, 0.04	ns	− 0.31	− 0.69, 0.07	ns	− 0.34	− 0.74, 0.05	ns
Infarct LGE	− 0.01	− 0.32, 0.32	ns	− 0.05	− 0.39, 0.29	ns	− 0.07	− 0.42, 0.28	ns
Hs-cTnI				0.01	− 0.10, 0.11	ns	0.01	− 0.09, 0.12	ns
BNP				− 0.02	− 0.11, 0.07	ns	− 0.01	− 0.10, 0.09	ns
Age per 10 years							− 0.03	− 0.14, 0.09	ns
Male							0.06	− 0.20, 0.33	ns

Zva, iECV, hs-cTnI and BNP were log_2_-transformed

*Zva* aortic impedance, *iECV* indexed extracellular volume, *LGE* late gadolinium enhancement, *hs-cTnI* high sensitive cardiac troponin I, *BNP* brain natriuretic peptide

### Prediction of events by CMR EF1

**Fig. 4:**
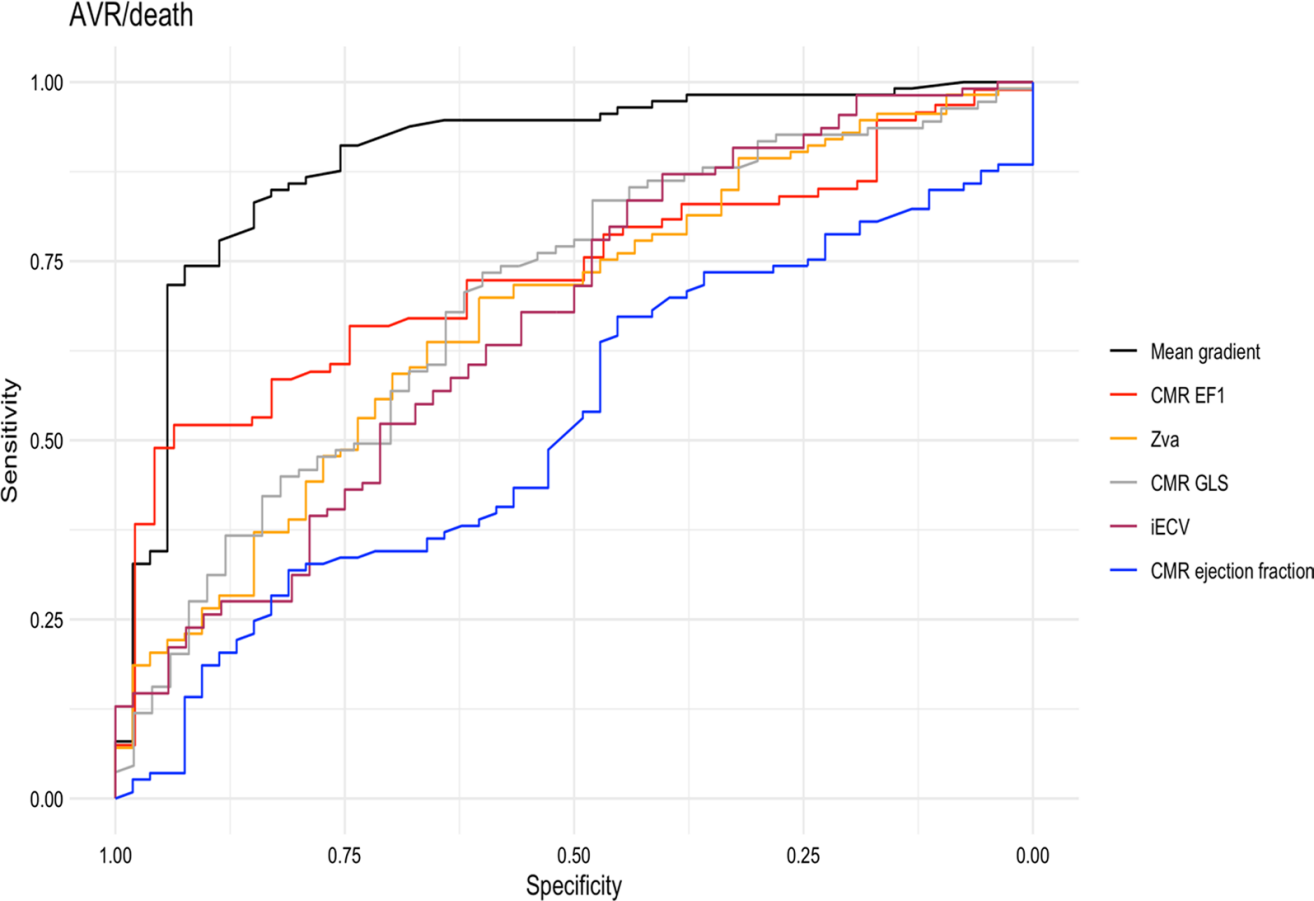
ROC curve for prediction of events. *EF1, first-phase* ejection fraction, *GLS* global longitudinal strain, *Zva* aortic impedance, *iECV* indexed extracellular volume

**Fig. 5:**
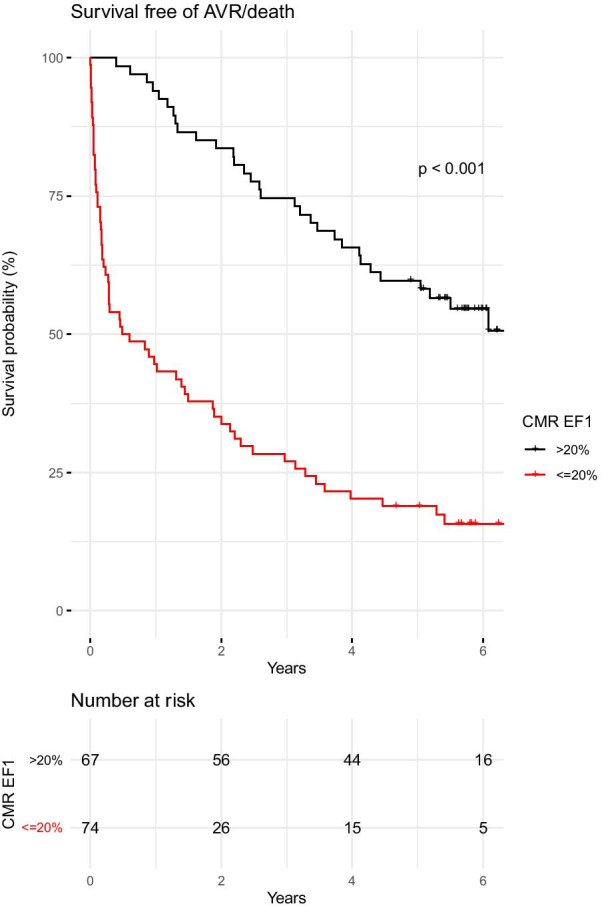
Kaplan–Meier Curve according to CMR EF1 (cut off value: 20%) for prediction of aortic valve replacement (n = 78) or death (n = 16)

ROC curve analyses (Fig. 4) demonstrated that mean aortic valve gradient had the largest area under the curve (AUC, 0.88). CMR EF1 (0.73) was a better discriminator of the primary outcome than other functional parameters, including CMR GLS (0.69) and CMR EF (0.55). A threshold for CMR EF1 of 20% (defined by our previously obtained threshold of 25% by TTE, adjusted for the 5% mean difference between CMR EF1 and TTE EF1) yielded a sensitivity of 74% and a specificity of 66%. Kaplan–Meier analysis (Fig. 5) showed that the probability of event-free survival was lower in those patients with a low CMR EF1 (Fig. 5, log-rank *p* < 0.001). On univariate and multivariable Cox regression analysis, CMR EF1 was a strong predictor of the primary outcome (Table 4). The adjusted hazard ratio for CMR EF1 was 0.93 (95% confidence intervals 0.89–0.97, *p* = 0.001) per % change in EF1. When the analysis was restricted to patients with severe AS, the hazard ratio for CMR EF1 was similar (0.90, 95% confidence intervals 0.86–0.94, *p* < 0.001) to that in the whole cohort. Patients with a low SV1/SV (defined as <  = 34%, on ROC analysis) had a lower rate of event-free survival compared to those with a high SV1/SV (log-rank p = 0.04). There was no significant difference in the prediction of outcome by CMR and TTE EF1. iECV was associated with outcome on univariate analysis but not after adjusting for CMR EF1 (Table 4) [13].

**Table 4:** Univariate and multivariate Cox regression analysis in patients with both CMR and TTE EF1 (n = 126)

	HR	CI (95%)		*P*	HR	CI (95%)	*P*	HR	CI (95%)	*P*
	Univariate				Multivariate CMR EF1			Multivariate TTE EF1		
Age	1.01	0.99, 1.03	0.25		1.01	0.82, 1.23	> 0.9	1.01	0.82, 1.24	> 0.9
Male	1.30	0.70, 2.40	0.41		1.59	0.93, 2.74	0.091	1.20	0.70, 2.04	0.5
NYHA										
II	1.19	0.77, 1.83	0.43		0.95	0.54, 1.66	0.9	1.06	0.60, 1.86	0.9
III/IV	3.43	2.16, 5.47	** < 0.001**		2.41	1.29, 4.51	**0.006**	3.01	1.56, 5.82	**0.001**
MPG	1.05	1.04, 1.06	** < 0.001**		1.06	1.04, 1.08	** < 0.001**	1.07	1.04, 1.09	** < 0.001**
CMR EF1	0.89	0.86, 0.92	** < 0.001**		0.93	0.89, 0.97	**0.001**	**-**	**-**	**-**
TTE EF1	0.88	0.85, 0.91	** < 0.001**		-	-	-	0.89	0.86, 0.93	** < 0.001**
Infarct LGE	0.54	0.32, 0.91	**0.02**		1.06	0.58, 1.94	0.8	0.55	0.29, 1.05	0.068
iECV	1.06	1.03, 1.09	** < 0.001**		0.99	0.95, 1.02	0.5	0.99	0.96, 1.03	0.6

*HR* hazard ratio, *CI* confidence interval, *NYHA* New York Heart Association Class, *MPG* mean pressure gradient, *CMR* cardiac magnetic resonance, *EF1* first-phase ejection fraction, *TTE* transthoracic echocardiography, *LGE* late gadolinium enhancement, *iECV* indexed extracellular volume

## Discussion

To our knowledge, this is the first study to derive CMR EF1. Previous studies have demonstrated TTE EF1 to be a strong predictor of major cardiac events in AS patients[1, 2]. Consistent with our previously published TTE data in this cohort, we found that CMR EF1 fell with increasing AS severity, had similar associations with assessments of LV afterload, myocardial fibrosis and outcomes, but had less variability than TTE EF1. CMR EF1 was lower (mean difference: -5.0 ± 7.8%) than by TTE. There was modest correlation between CMR EF1 and TTE EF1 (r = 0.51, *p* < 0.001). However, CMR EF1 and TTE EF1 were obtained on different occasions and therefore could have been influenced by both physiological variation and variation inherent in the measurement technique. With regard to the latter, CMR is regarded as the gold-standard for volumetric measurements but has limited temporal resolution, whereas TTE has greater temporal resolution but suffers from sub-optimal endocardial definition. Our data demonstrated that, compared to TTE, CMR is associated with an approximate halving of the variability of EF1 measurement suggesting that precision in volumetric assessments had a greater influence than that of temporal resolution. This suggests that CMR is the preferred modality for the measurement of EF1. However, it is likely that technological improvements to both modalities will improve EF1 precision. In particular, for CMR, higher temporal resolution achieved through a greater number of phases per cardiac cycle or through more sophisticated post-processing to define the time of peak aortic flow is likely to improve precision further. It is possible that other measures of early systolic function might perform as well or better than EF1. However, in the present study SV1/SV, which theoretically should provide a measure of EF1/EF and can be derived from phase contrast measurements alone correlated poorly with EF1/EF and was a relatively poor predictor of outcome. This may be due to errors in measurement of flow, limited temporal resolution of aortic flow or the presence of mild mitral regurgitation.

In the present study, where the majority of clinical events were AVR, MPG was, as expected, the most important predictor of outcome. However, CMR EF1 was a powerful predictor of AVR or death with a threshold of 20%, independent of AS severity. This is consistent with our TTE EF1 data[1, 2], allowing for the mean difference of 5% between CMR EF1 and TTE EF1. The prognostic value of EF1 demonstrates the importance of examining LV function in early systole over the portion of the cardiac cycle in which there is active contraction. The marked discordance between EF1 and overall LVEF in relation to outcomes is consistent with length-dependent regulation of sarcomere function acting as a homeostatic mechanism to preserve EF when initial contraction is impaired.

CMR allows the correlation of systolic LV function with structural change in the myocardium. That iECV was a predictor of outcome on univariate but not on multivariable analysis once EF1 was included in the model suggests that impact of iECV on outcome is mediated through its functional effects on early systolic contraction. This would be consistent with early expansion of the extracellular matrix due to chronic increased afterload as well as later apoptosis of myocytes and replacement fibrosis, both of which may occur prior to the onset of symptoms or a deterioration in overall ejection fraction [14, 15]. The combined power of CMR to assess early systolic function by EF1 as well as the underling myocardial structure may offer new insights into a variety of cardiac pathologies characterised by myocardial fibrosis and help refine the structural measures most closely related to function and hence to prognosis.

## Limitations

The present study has several limitations. Firstly, this is a post-hoc analysis of a single centre observational cohort with a relatively small sample size. Our observations regarding EF1 are consistent with two prior reports of TTE EF1 in AS, one of which was conducted in this cohort, but are subject to confounding. Conclusions regarding causality cannot be drawn from the cross-sectional correlations presented. Both CMR and TTE EF1 were measured from a single beat, which did not account for beat-to-beat variation, a weakness common to many haemodynamic measurements. Phase contrast (flow) and cine imaging were not obtained at the same time and this could have contributed to variation in EF1. However, the majority of patients were in sinus rhythm and variability of CMR-derived EF1 was less than that of TTE-derived EF1. CMR and TTE were not performed on the same day but in most subjects were performed within 5 days. CMR EF1 requires adequate short axis 3D stack image quality and could not be measured in 25 patients. Larger prospective studies will be required to determine the relative prognostic power of CMR and TTE EF1.

## Conclusions

EF1, a simple and robust marker of early LV impairment, be measured accurately by CMR, and predicts outcome in AS. Its measurement by CMR is more reproducible than that by TTE and may facilitate LV structure–function analysis.

## Data Availability

The datasets used and/or analysed during the current study are available from the corresponding author on reasonable request.

## Abbreviations

AS: Aortic stenosis
AVA: Aortic valve area
BNP: Brain natriuretic peptide
CMR: Cardiovascular magnetic resonance
ECV: Extracellular volume fraction
EDV: End-diastolic volume
EF: Ejection fraction
EF1: First-phase ejection fraction
GLS: Global longitudinal strain
hs-cTnl: High-sensitivity cardiac troponin I
LGE: Late gadolinium enhancement
LOA: Limit of agreement
LV: Left ventricle/left ventricular
LVMI: Left ventricular mass index
MPG: Mean pressure gradient
NYHA: New York Heart Association
ROC: Receiver-operating characteristic
SVI: Stroke volume index
TTE: Transthoracic echocardiography
V1: Volume at the time of peak aortic flow
Zva: Valvuloarterial impedance
